# Return of fertility after discontinuation of contraception: a systematic review and meta-analysis

**DOI:** 10.1186/s40834-018-0064-y

**Published:** 2018-07-23

**Authors:** Tadele Girum, Abebaw Wasie

**Affiliations:** Department of Public Health, College of Medicine and Health Sciences, Wolkite University, Wolkite City, Ethiopia

**Keywords:** Return of fertility, Contraceptives, Intrauterine device, Implants, Pills

## Abstract

**Introduction:**

Along with increasing availability and utilization of contraception, It is also important to confirm that the effects of contraception use on resumption of fertility after discontinuation However currently evidences on resumption of fertility after contraception use are inconclusive and practically fertility after termination of contraception remains a big concern for women who are using contraception. This fear poses a negative impact on utilization and continuation of contraception. Therefore, Estimating the rate of pregnancy resumption after contraceptive use from the available reports and identifying the associating factors are important for designing a strategy to overcome the problem.

**Methods:**

The review was conducted through a systematic literature search of articles published between 1985 and 2017. Five bibliographic databases and libraries: PubMed/Medline, Global Health Database, Embase, the Cochrane Library, and African Index Medicus were used. After cleaning and sorting, analysis was performed using STATA version 11. The pooled rate of conception was estimated with a random-effects model. Heterogeneity was assessed by the I^2^ and publication bias through funnel plot.

**Results:**

Twenty two studies that enrolled a total of 14,884 women who discontinued contraception were retained for final analysis. The pooled rate of pregnancy was 83.1% (95% CI = 78.2-88%) within the first 12 months of contraceptive discontinuation. It was not significantly different for hormonal methods and IUD users. Similarly the type of progesterone in specific contraception option and duration of oral-contraceptive use do not significantly influence the return of fertility following cessation of contraception. However the effect of parity in the resumption of pregnancy following cessation of contraception was inconclusive.

**Conclusion and recommendation:**

Contraceptive use regardless of its duration and type does not have a negative effect on the ability of women to conceive following termination of use and it doesn’t significantly delay fertility. Therefore, appropriate counseling is important to assure the women to use the methods as to their interest.

## Background

Wide ranges of effective and safe reversible modern contraceptives are available in the contemporary world. Despite the advancement in contraceptive technologies and organized international effort over the last few decades; the concern of women who use reversible contraception related to time to return of fertility still remained unanswered [1–3]. Most contraceptives have been modified to improve their safety and tolerability without compromising efficacy. It is also important to know the effect of contraception use on the subsequent fertility [1, 3].

However, currently evidences regarding resumption of pregnancy after contraceptive discontinuation are inconclusive. Delay of fertility after termination of contraception remains a big concern for women who are using contraception. Particularly women who ever experienced post pill amenorrhea or fail to become pregnant within expected date of fertility after termination of contraception have speculated contraceptive options cause delayed return of fertility.

Controlling unwanted fertility with highly effective reversible contraception allowed couples to have the number of children they want at the time they want to have. On the other hand fertility delay or impairment as a result of prior contraception use may lead to dissatisfaction and lower contraception use irrespective of actual desire [3–7].

Approximately 15% of couples experience infertility (fail to get pregnant within 1 year) [6], women who use hormonal contraception have considerable concern of delayed or impaired fertility upon discontinuation. Delayed return of fertility or infertility among previous contraceptive users is commonly linked to their contraceptive use. Therefore, this premise that leads to misconception among family planning users need to be synthesized and tested using the available evidences across the globe.

These concerns were also raised by scholars from early reports that Oral Contraceptive use may cause secondary amenorrhea, which is associated with anovulation and reduced reproductive fecundity. IUD may also cause infertility secondary to pelvic inflammatory disease (PID) [7]. It was believed that exogenous hormonal therapy causes delayed return of normal function of hypothalamic/pituitary/ovarian axis [8–11] and temporary infertility [12]. However these concerns were disproved from more recent studies partly from development of low dose hormonal contraception, prevention of PID and implementation of scientific technique [13–16].

There are a number of studies and few specific reviews conducted to assess the effect of different forms of contraceptives on subsequent pregnancies. The findings were inconclusive, in some studies contraception shown to have only an initial (temporary) delay in conception for the first few months after discontinuation [13–16]. While in recent studies no association was observed between contraceptive use and secondary amenorrhea [17–19] except with higher doses of oestrogen [20]. On the other hand many studies have reported that, the type of intrauterine device as well as duration of use has not been found to be related to fertility return [21].

Therefore, we aimed to conduct a comprehensive systematic review and meta-analysis through reviewing globally published observational studies on the effect of fertility return after discontinuation of different contraception among married and in union. Return of fertility is measured in terms of pooled rate of fertility return within 1 year in order to bring conclusive evidence. So that policy makers and other stakeholder could have synthesized evidence to rely on in decision making on prospect of the problem.

## Methods and material

### Literature search strategy

This systematic review and meta analyses is conducted according to PRISMA (Preferred Reporting Items for Systematic Reviews and Meta-Analyses) guideline [22]. Systematic literature search of articles was made. Articles published between 1985 and 2017 containing information on rate of pregnancy following cessation of reversible contraception were retained for systematic review and meta-analysis. Electronic bibliographic databases and libraries including PubMed/Medline, Global Health Database, Embase, the Cochrane Library, and African Index Medicus were used to retrieve published articles. Combination of search terms were used with (AND, OR, NOT) Boolean (Search) Operators. (1: contraception discontinuation; 2: IUD discontinuation; 3: Implant discontinuation; 4: Hormonal contraception discontinuation; 5: 1 or 2 or 3 or 4; 6: return to fertility; 7: rate of fertility; 8: time to pregnancy; 9: planned pregnancy; 10: fecundity; 11: delay in conception; 12: time to conception; 13: pregnancy delay; 14: time to ovulation, 15: 6 or 7 or 8 or 9 or 10 or 11 or 12 or 13 or 14; 16: 5 and 15). In addition the reference lists of primary and pertinent review articles were also uploaded into an EndNote XI library (EndNote, Carlsbad, CA, USA) to identify cited studies not captured by the electronic search and after all checked for duplications.

### Study selection process and eligibility criteria

Studies published in English language anywhere in the world that reported a 12 month pregnancy rates following discontinuation of a reversible contraceptive method with the intention to get pregnant, with sample size of 100 women and above, and prospective clinical/observational study designs were the inclusion criteria for this systematic review and meta-analysis. One-year pregnancy rate was used to exclude women who developed secondary infertility. Unable to conceive despite of unprotected sexual intercourse with optimum frequency for 1 year and above is called infertility, but the scope of this review is delayed return of fertility after cessation of contraceptive use. Therefore, 1 year rate of pregnancy is more informative to assess delayed resumption to fertility than other time scales. The outcome of interest was the rate of pregnancy among modern reversible ex-contraception users. However studies reporting rate of delivery as the only outcome, Studies published before 1985, studies assessing fertility after abortion or post abortion contraception were excluded. Also Studies conducted in the same location during the same time period were considered as potential duplicates and therefore excluded from the analysis. Three experts reviewed each article and decided based on the inclusion and exclusion criteria.

### Data extraction and abstraction

Titles and abstracts derived through primary electronic search were thoroughly assessed for possibility of reporting pregnancy rates within 1 year period and filtered for potential eligibility. If needed, and whenever possible, the authors were contacted for clarifications. From each eligible research, the following information was extracted based on the preformed database (Excel, Microsoft, 2010) format: about author, study participants, studies (study design, sample size, study setting), Type of contraception, length of use, year of publication, year of study start and end, eligibility criteria, rate of pregnancy, etc. All data were extracted independently and in duplicate using a standardized extraction form. Returned abstracts were reviewed and full texts retrieved if they contained relevant information. At the same time, each selected research was assessed for methodological quality and possibility of bias. The outcome variable (rate of pregnancy) was defined as the proportion of women who were pregnant within 1 year of contraception discontinuation. The effect size is measured in rates/proportion.

### Assessment of risk of bias in individual studies and across studies

Risk of bias in individual studies and across studies was assessed through evaluating reliability and validity of data for each important outcome variables. Methods used to assess the outcome variable in each study were also used to assess risk of bias. For all studies; the study design, study participants, the outcome, presence of loss to follow up were assessed based on the eligibility criteria and quality assessment check list. Moreover all studies were prospective studies which employed the same participants and outcome was measured in the same standard. The risk of publication bias and heterogeneity was assessed through the standard statistical approaches. However there are still uncontrolled biases at the selection of study participants, analysis of the result and presentation/publication of the report.

### Data analysis

After cleaning and sorting the final database was exported into STATA 11.0 for analysis (STATA, College Station, TX, USA). An outcome of interest was rate of pregnancy after discontinuation of contraception before or at 12th month. Estimate of pregnancy rate was assessed for each study and standardized mean with 95% confidence interval was used. These were calculated with a random effects model according to the DerSimonian and Laird method [23]. Heterogeneity was assessed by the I^2^ and values greater than 50% considered representing significant heterogeneity. When heterogeneity between studies was found to be significant, pooled estimates were based on random-effect models and the Hedges method of pooling. Results were displayed visually in forest plots. Bias was investigated by construction of funnel plots and Analysis was performed using the ‘metan’ and related functions in STATA version 11 (College Station, TX).

## Results

### Studies included

**Fig. 1:**
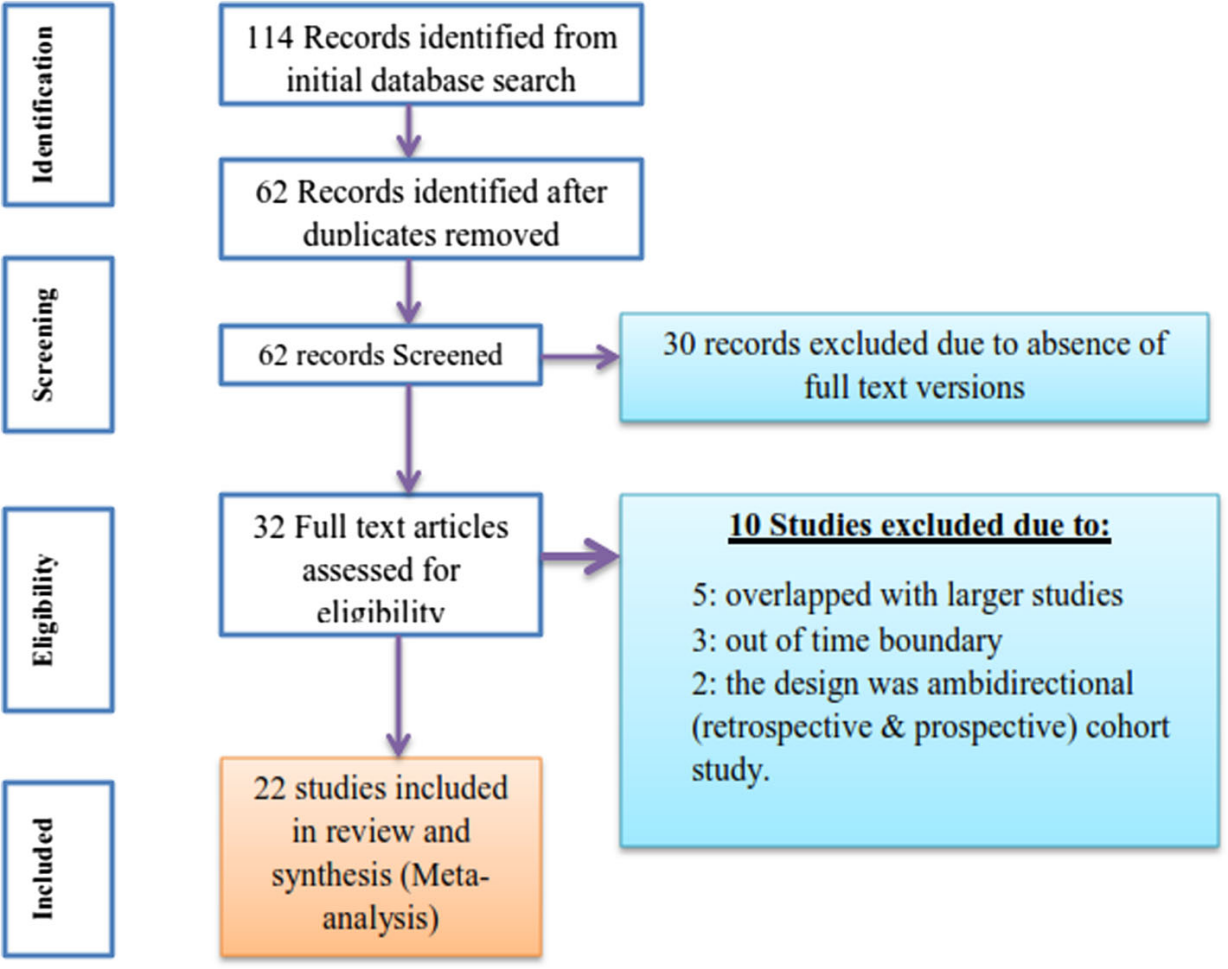
Flow chart for study search, selection and screening for the review

From 114 studies initially identified, 22 [24–44] were retained for final analysis based on the inclusion and exclusion criteria and quality assessment. From the initial search, literatures were identified as abstract, bibliography and full text research from the selected electronic data bases. After reviewing the abstracts, 62 possible researches were transferred to preformed format of endnote, searched for full text research and cleaned for duplications and 32 abstracts of articles were identified for full text review. Of these reviewed in full text, 30 were removed prior to analysis for different reasons: 5 studies overlapped with larger studies, 3 studies were out of time boundary and for 2 studies the design was ambidirectional (retrospective & prospective) cohort study (Fig. 1).

### Description of findings

The studies enrolled a total of 14,884 women who discontinued contraception for the sake of pregnancy. Of them 735 discontinued implants, 139 discontinued injectable contraception, 2374 women discontinued IUD and 11,636 discontinued oral contraception. The primary outcome of the studies was rate of pregnancy after discontinuation of contraception at 12 month. Some studies also assessed the possible reasons for delay in resumption of pregnancy. With duplicates, 5 studied implants [24–29], 2 injectables [30, 31], 5 oral contraception [32–36] and the rest 12 assessed return of fertility after discontinuation of IUD [37–43]. All included studies were prospective cohort and prospective observational designs conducted in different parts of the world and published between 1985 and 2017 with English language.

The mean exposure time (duration of use) of implant users extend from 29.1-55.8 months, injectable users have exposure time of 21.3-35.7 months, while oral contraception users were exposed for 24-84 months and IUD users retained for 19-28 months. Survey characteristics are described in (Table 1).

**Table 1:** Characteristics of studies included in the review

Authors & FP method	Publication year	Type of contraception	Sample size	Duration of use	Pregnancy rate	Design of the study	Setting
Implants							
[24] Affandi et al.	1999	Implanon	80	mean 35.3 month	48.80	Prospective	Indonesia
[24] Affandi et al.	1999	Norplant	80	mean 55.8 month	37.50	Prospective	Indonesia
[25] Buckshee et al.	1995	Norplant II	159	30.0 ± 11.5 months	80.30	Prospective	India
[26] Sivin et al.	1992	Norplant II	116	Mean 29.1 month	84.00	Prospective obs.	Not specified
[26] Sivin et al.	1992	Norplant VI	62	Mean 33.6 ± 1.9	83.00	Prospective obs.	Not specified
[27] Singh et al	1989	Norplant	100		90.00	prospective	Singapore
[28] Affandi et al	1987	Norplant VI	51	32.4 ± 8.8 months	76.50	Prospective	Indonesia
[29] Diaz et al	1987	Norplant VI	87	1–8 years	85.6	Prospective	Chile
waited mean (12 month Return rate of pregnancy among ex-users of implants)					74.70		
Injectable							
[30] Bahamondes et al.	1997	Cyclofem-monthly	70	7.1 ± 4.6 cycle	82.90	Prospective	4 countries^a^
[31] ICMR task Force	1986	Norethisterone	69	11.9 ± 4.9 months	72.50	Prospective	India
Waited mean (12 month Return rate of pregnancy among ex-users of injectable)					77.74		
Oral contraceptives							
[32] Barnhart et al.	2009	Oralcontraceptives	21		81.00	Prospective	multicenter
[33] Cronin et al.	2009	Oralcontraceptives	2064	7.2 yrs	79.40	Prospective obs.	7 countries^b^
[34] Wiegratz et al.	2006	30 mcg EE/2 mgDNG	706	21.5 ± 16.8 cycle	94.00	Prospective obs.	Germany
[35] Farrow et al	2002	Oralcontraceptives	8497		88.00	Prospective	England
[36] Zimmermann	1999	31 mcg EE/2 mgDNG	348	med. 4-6 cycle	95.00	Prospective obs.	Germany
Waited mean (12 month Return rate of pregnancy among OC ex-users)					87.04		
Intra uterine device (IUD)							
[37] Delbarge et al	2002	GyneFix	128	mean104.6 ± 93wk	88.00	Prospective	Belgium
[38] Tadesse et al	1996	Copper T-200	780	med. 3.5 years	86.00	Prospective obs.	Ethiopia
[39] Anderson et al	1992	Nova-T	71	Median 21 month	71.20	Prospective	5 countries^c^
[39] Andersson et al	1992	LNG-20 IUS	138	Median 19 months	79.10	Prospective	5 countries
[26] Sivin et al.	1992	TCu380Ag	103	mean 38.9 months	77.00	Prospective obs.	Not specified
[26] Sivin et al.	1992	LNG-20 IUS	91	mean 30.2 months	84.00	Prospective obs.	Not specified
[40] Gupta et al.	1989	Copper plus Prog.	91	56% b2 years	92.30	prospective	Not specified
[28] Affandi et al	1987	Lippes C IUDs	75	31.8 ± 8.6 month	74.70	Prospective	Indonesia
[41] Skjeldestad et al	1987	Copper IUDs	101	56% b24 months	81.00	Prospective obs.	Norway
[42] Belhadji et al.	1986	TCu380Ag	50	23.96 months	91.10	Prospective	Not specified
[31] *ICMR Task Force*	1986	Copper T-200	110	28.2 ± 14.7 months	83.60	Prospective	India
[42] Belhadji et al.	1986	LNG-20 IUS	60	22.72 months	96.40	Prospective	Not specified
[43] Randic et al	1985	all forms of IUD	576	59% b3 years	86.10	Prospective	Yugoslavia
Waited mean (12 month Return rate of pregnancy among IUD ex-users)					84.75		

*obs* observational, *FP* Family planning, *OC* oral contraception

^a^Brazil, Chile, Columbia, Peru

^b^Austria, Belgium, Denmark, France, Germany, Netherlands, UK

^c^Denmark, Finland, Hungary, Norway, Sweden

The 12 month pregnancy rate following discontinuation of different forms of implant with the intension to have pregnancy was measured in 8 studies (with duplicates). Based on this estimates, 74.7% of ex-implant users get pregnant within 12 months. Moreover a study reporting exceptionally lower rate of pregnancy (48.8% among Implanon and 37.5% among Implant II-VI) [24] is removed, the mean weighted pregnancy rate increases to 83.45%. With the same hormonal composition ex-injectable contraception users have a pregnancy rate of 77.74% which is estimated only with two studies and ex-oral contraception users have a pregnancy rate of 87.04**.**

One year pregnancy rate following cessation of different types of Intrauterine device (IUD) was 84.75%, which is weighted among 13 studies (with duplicates). The studies also noted that there is no significant difference between different types of IUD in terms or fertility resumption. Also in this case pregnancy was resumed with in a brief period of time following cessation of use or removal of the device.

The highest rate of pregnancy among implant users of 90% was reported by Singh et al. [27] in Singapore, while the highest resumption rate of 95% following cessation of oral contraception was reported by Zimmermann et al. [36] among German women and as high as 96.4% of ex-IUD users in Belhadji et al. [42] report were conceived within 1 year. There is a wide overlap in the reported 1-year pregnancy rates after discontinuation of different forms of contraception. The rate of pregnancy was unexpectedly higher among ex-oral contraception users, followed by IUD users. However this difference was not statistically significant.

### Pooled estimates and tests

Heterogeneity tests showed significant variations between studies (Q = 611.5, *P* = 0.000), where Q-value, the weighed sum of squares on a standardized scale was significantly different compared with expected weighed sum of squares and I-squared showed that 95.3% of the observed dispersions are attributed to real rather than spurious variations. Also the funnel plot showed evidence of bias with some of the studies missing at the bottom rather than around the main effect. Accordingly, the Duval and Tweedie’s trim and fill test was applied to adjust for the publication bias.

**Fig. 2:**
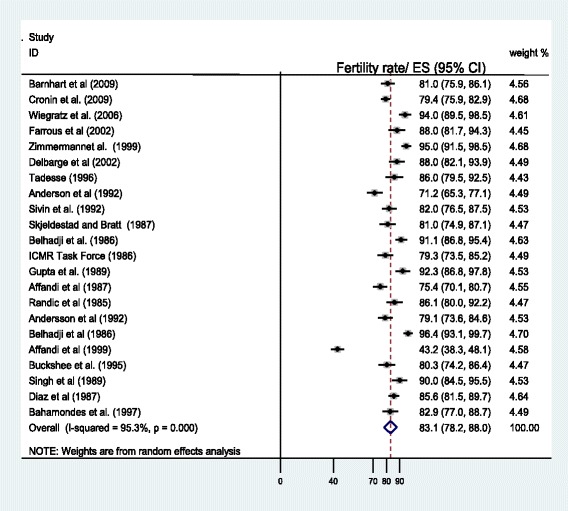
Forest plot showing the rate of one year pregnancy following discontinuation of contraception, weighted according to random-effects model

The presence of heterogeneity and publication bias resulted in adjustment of the point estimate of the rate of pregnancy following cessation of different types of contraception under a random effect model from 84.36 to 83.1%. In a fixed effect model the pooled estimate of pregnancy rate was 84.36% (95% CI = 83.3-85.4%) and in a random effect model the pooled estimate of pregnancy rate became 83.1% (95% CI = 78.2-88%), while estimates of each study are unchanged. In all cases pooled estimate from random effect model was used for report and discussion (Fig. 2).

The effect of some demographic characteristics, like age at contraceptive discontinuation and parity on resumption of pregnancy were inconclusive from these findings. Some studies reported a decrease in resumption of pregnancy rates with increasing age [25, 26, 33, 37, 38, 41, 43] and others showing no such decrease with increased age [30, 34, 40, 42]. In addition, the effect of parity on pregnancy rates was inconsistent, with some studies suggesting that age-adjusted pregnancy rates were significantly higher in multiparous women [33], while in another study it was lower among nulliparous women [41], unaffected by parity [26] and significantly lower among multiparous women in Delbarge et al. [37] report. The possible effect of hormonal contraception and prolonged use of oral contraception on impaired fertility was not supported in these findings [25–31]. Higher level of fertility was observed among non-hormonal contraception users in many studies [37–43] however the difference was not statistically significant.

## Discussion

According to this review 83.1% (95% CI = 78.2-88%) of women who discontinued contraception became pregnant within the first 12 months. Return of fertility at the first year was not significantly different for hormonal methods and IUD users. Similarly type of progesterone in contraception and duration of oral-contraceptive use do not significantly influence return of fertility following cessation of contraception. However effect of parity in resumption of pregnancy following cessation of contraception was inconclusive.

The rate of fertility return in this review was comparable to other reports of reviews and articles which assessed specific types of contraception [45–48]. The rate of pregnancy for oral contraceptives, copper IUDs and the LNG-IUS ex-users was also overlap with each other and comparable to previous findings. However the finding was slightly lower than reports of women who discontinued barrier methods or using no contraceptive method of 85.2– 94% [49, 50]. This difference may be due to the fact that hormonal contraceptives commonly take months to clear from the body which results in temporary delay in resumption of pregnancy for months [44, 47, 48].

One year pregnancy rate of (37.5-90%) following cessation of Implant also overlaps across different studies [45, 46]. There are studies which report exceptionally low rate of pregnancy within 1 year after cessation of contraception as evidenced by Affandi et al. [24] which reports (37.5 and 48.8% for Norplant and Implanon ex-users respectively). However when the study with low rate of pregnancy after cessation of contraception is removed the rate of fertility return is comparable to other methods. Moreover, no significant difference was reported between different forms of Implants. This may be explained by the fact that implants are impregnated with similar hormone which doesn’t create a difference.

Return of fertility following termination of IUD was not compromised at all and resumption ranges between (71-96%) with a mean of 84.75%. Moreover, type of IUD and duration of use as well as addition of hormones to the device do not compromise pregnancy [37–43]. In line with this finding Mansour et al. [45] reported pregnancy rate of 86.1 to 92.3% following termination of IUD which is comparable to natural method users and non-users. This finding also witnesses that prompt resumption of fertility after termination of IUD. As explained by other findings type and duration of IUD use doesn’t matter the rate of pregnancy after cessation [46, 47].

It is commonly believed that oral contraception may compromise conception, however, this review reported higher rate of conception (87%). In line with this finding other researchers reported comparable return of fertility after cessation of oral contraception [47–50] However, this review and meta-analysis appreciate presence of brief delay in return of fertility after cessation of hormonal contraception use until the bioavailability of the hormone in blood is completely cleared. It is also noted that the 3 months hormonal contraception use hinders pregnancy, but the effect is extremely low for12 month users and no effect for 24 month users [32–36, 47–49]. The Concern of impaired fertility which was reported with high-dose of oral contraceptive pills in early years is not a problem currently. This is due to presence of low dose contraception regimen for use [33, 34, 48].

Our review also shows that the duration of contraception use was not significantly affected with return of fertility. It is in line to many studies included in the review [24–44] and the report of some other researches [45–49]. On the other hand there are evidences which narrate women who used oral contraceptives for a longer duration may had a slightly lower rate of pregnancy than did women using oral contraceptives for a shorter period of time [44] which could be the effect of age, in which fertility decreases as age advances.

However, since none of the studies were randomized control trials and most of the studies had small sample sizes, the possible relationship between extended use of hormonal contraception and the rate of resumption of pregnancy may not come across through appropriate and reliable conclusion.

Similarly our review showed that the progestin type had no major effect on the rate of pregnancy over the short term and long-term. This is because rather than duration, dose matters. However currently only low dose preparations are in use. Therefore, delay in fertility may not be common following termination of contraception use. In addition, return of fertility among women discontinuing extended or continuous OC regimens is similar to that observed with cyclic OCs [32–36]. This result was also reported from previous reviews assessing the return of fertility following cessation of oral contraception [45–50].

The effect of parity on the rate of fertility was inconclusive. The finding of this review shows that parity may or may not enhance fertility. Studies included in the review particularly compared nulliparous and multiparous women which ignored the rate of infertility [44]. Therefore, higher rate of pregnancy among multiparous women who are proved to be fertile are expected. In all cases baseline prevalence of infertility may influence fertility rates of women seeking pregnancy following discontinuation of a contraceptive method.

## Conclusion and recommendation

Resumption of fertility following cessation of contraception was not affected by use of contraception, type of contraception, duration of use and type of progesterone. However the effect of parity in the resumption of pregnancy following cessation of contraception was inconclusive. Therefore, it is important to counsel women that prior contraceptive use regardless of its duration and type does not have a negative effect on subsequent fertility, so that they can choose and use the duration they want.

## Abbreviations

FP: Family planning
IUD: Intrauterine device
LARC: Long acting reversible contraception
MeSHs: Medical subject headings
PRISMA: Preferred reporting items for systematic reviews and meta-analyses
