# Correction: Return of fertility after discontinuation of contraception: a systematic review and meta-analysis

**DOI:** 10.1186/s40834-023-00226-y

**Published:** 2023-04-21

**Authors:** Tadele Girum, Abebaw Wasie

**Affiliations:** Department of Public Health, College of Medicine and Health Sciences, Wolkite University, Wolkite City, Ethiopia

**Correction:** ***Contracept Reprod Med*3, 9 (2018)**

10.1186/s40834-018-0064-y

Following publication of the original article [1], it was reported that the Abstract section should be updated to remove the grammatical errors.

The correct Abstract should read:

Along with increasing availability and utilization of contraception, it is also important to confirm the effects of contraception use on resumption of fertility after discontinuation. However, current evidence on resumption of fertility after contraception use is inconclusive and practical fertility after termination of contraception remains a big concern for women who are using contraception. This fear poses a negative impact on the utilization and continuation of contraception. Therefore, estimating the rate of pregnancy resumption after contraceptive use from the available reports and identifying the associating factors are important for designing a strategy to overcome the problem.

The original article [1] has been updated.
